# Tricuspid Annulus Dilation in Patients With Combined Functional Tricuspid Regurgitation and Left-Heart Valvular Disease: Does Septal Annulus Not Dilate?

**DOI:** 10.3389/fcvm.2022.889163

**Published:** 2022-04-26

**Authors:** Peng Teng, Xiaoyi Dai, Yu Zou, Shuai Yuan, Yan Chen, Liang Ma, Yiming Ni

**Affiliations:** ^1^Department of Cardiovascular Surgery, The First Affiliated Hospital, College of Medicine, Zhejiang University, Hangzhou, China; ^2^College of Medicine, Zhejiang University, Hangzhou, China; ^3^Department of Echocardiography and Vascular Ultrasound Center, The First Affiliated Hospital, College of Medicine, Zhejiang University, Hangzhou, China

**Keywords:** tricuspid annular dilation, functional tricuspid regurgitation, tricuspid annulus (TA), tricuspid repair, tricuspid valve annuloplasty

## Abstract

**Background:**

This study aimed to investigate the course of tricuspid annulus dilation in functional tricuspid regurgitation with varied severities by direct intraoperative assessment.

**Methods:**

A total of 317 patients who underwent left heart surgery and concomitant tricuspid repair were divided into three groups according to the severity of the functional tricuspid regurgitation (mild, moderate and severe). Demographic and echocardiographic data were collected. The length of each tricuspid annulus segment was measured intraoperatively. The risk factors for preoperative severe functional tricuspid regurgitation and its postoperative recurrence were identified, and the impact of each tricuspid annulus segment on postoperative recurrence was compared.

**Results:**

In the course of tricuspid annulus dilation, the posterior annulus dilated 17% (group 1: 33.31 ± 6.94 mm vs. group 2: 35.56 ± 7.63 vs. group 3: 38.98 ± 8.70, *p* < 0.01), the anterior annulus dilated 13.4% (group 1: 36.71 ± 6.30 mm vs. group 2: 38.21 ± 8.35 vs. group 3: 41.63 ± 9.20, *p* < 0.01), and the septal annulus dilated 11.4% (group 1: 38.11 ± 5.28 mm vs. group 2: 39.76 ± 6.90 vs. group 3: 42.46 ± 7.50, *p* < 0.01). Tricuspid annulus circumference index (*p* < 0.01) independently correlated with preoperative severe tricuspid regurgitation and postoperative recurrence. When patients were grouped based on the length of each segment, the septal annulus demonstrated significantly higher sensitivity (*p* < 0.001) to postoperative recurrence than the anterior (*p* = 0.085) or posterior annulus (*p* = 0.262).

**Conclusions:**

This study revealed that each segment of tricuspid annulus could dilate in functional tricuspid regurgitation and highlighted the potential benefits of septal annulus plication in tricuspid annuloplasty, which may aid in the development of a methodology for prosthetic ring annuloplasty.

## Introduction

Functional tricuspid regurgitation (FTR) is the most common pathology involving the tricuspid valve, which generally occurs secondary to left-sided heart disease, atrial fibrillation (AF), or pulmonary hypertension as a consequence of tricuspid annulus (TA) dilation. Recently, the research is focused on the treatment of FTR (1), as it may deteriorate patients' long-term survival (2, 3).

Prosthetic ring annuloplasty plays an important role in tricuspid repair (4), which is designed based on the principle first reported by Deloche that the TA dilation occurs primarily in the posterior and anterior segments of the TA, and the septal annulus is relatively spared (5, 6). Based on this property, Carpentier proposed a technique for selecting the appropriate size of the tricuspid ring based on the length of the septal annulus as well as the area of the anterior leaflet (7). According to the manufacturer's protocol, the marker designs on the tricuspid rings indicate the corresponding positions of the anteroposterior and septoposterior commissures. However, these markers vary among the prosthetic rings (8), which suggests that the gold standard technique for tricuspid annuloplasty remains controversial.

Furthermore, when we selected the ring size during tricuspid annuloplasty based on the length of the septal annulus, we observed that Carpentier's technique of measurement was not applicable in some cases because the septal annulus could be larger than the largest size of the obturator (Supplementary Figures 1A–D). Several Japanese studies also confirmed our findings that the septal annulus could dilate in FTR to as large as 60 mm (9, 10), and markers on the annuloplasty ring do not always conform to the commissures due to a varied number of scallops in the posterior leaflet (11, 12). Moreover, Pfannmuller et al. observed that ring dehiscence after tricuspid annuloplasty only occurred at the septal annulus (13). A possible explanation might be that the area of ring dehiscence might be the consequence of continuous dilation with increased shearing forces. All these findings suggested that the septal annulus could dilate during the course of TA dilation.

Based on these conflicting results, there is an urgent need for a better understanding of the changes in TA during its pathological course. Therefore, this study aimed to investigate the course of TA dilation in patients with FTR with varied severities using direct intraoperative assessment, which could contribute to developing a methodology for tricuspid annuloplasty.

## Methods

### Patient Selection

A total of 317 patients with left-heart valvular disease and FTR who underwent left-heart surgery and TA repair between August 2017 and October 2020 in our hospital were enrolled. The surgical indications for TA repair included either moderate and severe tricuspid regurgitation, mild tricuspid regurgitation with annular dilation of ≥40 mm (or ≥21 mm/m^2^), or mild tricuspid regurgitation with AF. Surgical procedures of the left heart included mitral and aortic valve repair, mitral and aortic valve replacement, combined mitral and aortic valve replacement. If necessary, Cox-Maze IV procedure and coronary artery bypass grafting (CABG) were simultaneously performed in patients with AF and coronary heart disease, respectively. Patients who previously underwent left heart surgery or had right coronary artery disease were excluded. All patients were followed up after discharge.

### Echocardiography

Preoperative and postoperative echocardiographic data of all patients were recorded using transthoracic or transesophageal echocardiography. The FTR severity was classified into four degrees based on the vena contracta (VC) width in the apical four-chamber plane view and was qualitatively graded as trivial (VC <2 mm, jet area <1 cm^2^), mild (VC <3 mm), moderate (VC <7 mm), and severe (VC ≥7 mm). Left ventricular ejection fraction and heart chamber diameters were assessed according to the American College of Cardiology/American Heart Association (ACC/AHA) guidelines (14). Pulmonary artery systolic pressure (PASP) was calculated from the FTR jet velocity using the modified Bernoulli equation and was combined with an estimation of the right atrial pressure. FTR recurrence was defined as the presence of moderate or severe FTRs at follow-up.

### Surgical Protocol and Intraoperative Assessment

The surgeries were performed via median sternotomy or right thoracotomy. In patients receiving median sternotomy, cardiopulmonary bypass was performed by arterial cannulation of the ascending aorta and vein cannulation for both the superior and inferior venae cavae. In patients who underwent right thoracotomy, cardiopulmonary bypass was established through the femoral artery and vein cannulation. Myocardial protection was performed through antegrade perfusion of cold blood cardioplegia or del Nido cardioplegia solution. The mitral and tricuspid valves were exposed through the left interatrial groove and right atrium pathways, respectively. The retractor was gently pulled to expose the entire tricuspid valve under cardiac arrest or on a beating heart with full-flow cardiopulmonary bypass. A silk suture was used to measure the length of the TA, and this suture was measured using a ruler. After the intraoperative assessment, tricuspid annuloplasty was performed using the Kay procedure, Edwards MC3 rigid ring (Edwards Life Sciences, Irvine, California, USA), and Sorin Sovering flexible ring (Sorin Biomedica Cardio, Saluggia, Vercelli, Italy) according to the manufacturers' protocol.

### Statistical Analyses

Categorical variables were expressed as the number and percentage of patients. Continuous data were presented as either means ± standard deviations if the variables were normally distributed or as medians with interquartile ranges (1st−3rd) if they were non-normally distributed. Fisher's exact test or chi-square test was used to compare categorical variables. The unpaired student's *t*-test, one-way analysis of variance, Mann–Whitney *U* test, and Kruskal–Wallis *H* test were used to compare continuous variables between groups. Factors correlated with preoperative severe FTR and postoperative recurrence of FTR were analyzed using binary logistic regression and Cox proportional hazards regression analysis. In univariate analysis, significant factors (*p* < 0.1) were included in the multivariate analysis, and forward conditional logistic regression was used to identify the risk factors. The validation of the prediction model was presented as receiver operating characteristic (ROC) curves. Kaplan–Meier (KM) survival analysis was performed to investigate the impact of the length of TA on postoperative FTR recurrence. All statistical analyses were performed using statistical package of social sciences (version 26.0; SPSS Inc., Chicago, Illinois, USA) and Prism 8.2.1 (GraphPad Software Inc., CA, USA) software, and a *p*-value of < 0.05 was considered statistically significant.

## Results

### Patient Characteristics

A total of 317 (132 male, 41.6%) patients with an average age of 57.67 ± 11.42 years were included in the study. They were divided into three groups according to the FTR severity (group 1, 139 patients with mild FTR; group 2, 126 with moderate FTR; group 3, 52 with severe FTR). Preoperative and operative data are summarized in Table 1. The demographic data, including the gender, height, weight, body mass index, and body surface area (BSA), were similar between the groups; however, patients in groups 2 and 3 were significantly older (group 1: 54.98 ± 10.46 years; group 2: 58.52 ± 11.80 years, group 3: 62.81 ± 11.07 years, *p* < 0.01). Patients in groups 2 and 3 had higher proportion of class III–IV of New York heart association (NYHA) classification; a greater proportion of AF; and higher levels of N-terminal pro-brain natriuretic peptide (NT-BNP), total bilirubin, and creatinine than those in group 1. In the analysis of echocardiographic parameters (Table 1), patients in groups 2 and 3 had significantly larger dimensions of the heart chamber and more dilated TA, PASP, and lower tricuspid annular plane systolic excursion (TAPSE). The etiologies of left heart disease were similar between the groups. The procedures of left heart surgery included combined mitral and aortic valve replacement (*n* = 107), mitral valve replacement (*n* = 167), mitral valve repair (*n* = 45), aortic valve replacement (*n* = 9), CABG (*n* = 4), and Cox-Maze IV (*n* = 5). The methods of TA repair included the Kay procedure and tricuspid annuloplasty using Sorin Sovering flexible ring and Edwards MC3 rigid ring. One patient each in group 2 and group 3 expired due to refractory heart failure on postoperative day 4 and cerebral hernia on postoperative day 1, respectively. The remaining patients were followed up until November 1, 2021, and the median follow-up time was 18.90 (11.39–26.03) months. FTR recurrence was observed in 27 patients (8.5%).

**Table 1:** Preoperative and operative data.

	**Overall (*n* = 317)**	**Mild FTR (*n* = 139)**	**Moderate FTR (*n* = 126)**	**Severe FTR (*n* = 52)**	***P*** **-value**
Male	132 (41.6%)	52 (37.4%)	54 (42.9%)	26 (50%)	>0.05
Age	57.67 ± 11.42	54.98 ± 10.46	58.52 ± 11.80^**^	62.81 ± 11.07^**^^#^	<0.01
Height, cm	162.29 ± 7.53	162.12 ± 7.05	162.37 ± 7.54	162.56 ± 8.82	>0.05
Weight, kg	60.36 ± 10.51	61.00 ± 10.79	59.90 ± 9.56	59.75 ± 12.03	>0.05
BMI, kg/m^2^	22.82 ± 3.05	23.10 ± 3.04	22.65 ± 2.92	22.49 ± 3.38	>0.05
BSA, m^2^	1.69 ± 0.17	1.69 ± 0.17	1.68 ± 0.15	1.68 ± 0.19	>0.05
**NYHA**					<0.01
I–II	149 (47.0%)	91 (65.5%)	50 (39.7%)	8 (15.4%)	
III	136 (42.9%)	41 (29.5%)	68 (54.0%)	27 (51.9%)	
IV	32 (10.1%)	7 (5.0%)	8 (6.3%)	17 (32.7%)	
NT-BNP	292 (136–576)	184.5 (118–328.25)	305.0 (155.5–700)^**^	550.5 (302.25–921.75)^**^	<0.01
Total bilirubin	14 (10–20)	12 (8.75–16)	14 (10–19)^**^	21.5 (12.75–30.25)^**^^##^	<0.01
Creatinine	73 (64–87)	70 (61–82)	73 (65–89)^*^	80.5 (71.5–101)^**^	<0.01
AF	203 (64.0%)	73 (52.5%)	83 (65.9%)	47 (90.4%)	<0.01
**Echocardiography**					
LVDd, mm	50.87 ± 9.28	49.30 ± 9.15	52.07 ± 8.82^*^	52.27 ± 10.28^*^	<0.05
LVDs, mm	33.79 ± 7.10	32.25 ± 6.31	35.19 ± 7.29^**^	34.63 ± 8.01^*^	<0.05
LA, mm	50 (44–58)	48 (43–56)	51 (45–58)^**^	52 (48–59.5)^**^	<0.05
EF, %	62 (58.5–67)	63 (59–68)	62 (56–66)	62 (58–68.5)	>0.05
FS, %	34 (31–38)	34 (32–38)	33 (31–37)	33 (30–38)	>0.05
RA S-L, mm	51.36 ± 11.94	45.03 ± 8.19	49.72 ± 10.11^*^	59.81 ± 13.02^**^^##^	<0.01
RA S-I, mm	66.61 ± 13.70	60.95 ± 11.89	63.94 ± 11.59	76.16 ± 13.87^**^^##^	<0.01
TV S-L, mm	38.02 ± 6.62	34.33 ± 5.25	37.92 ± 4.70^**^	45.39 ± 7.51^**^^##^	<0.01
TV A-P, mm	38.01 ± 6.04	35.96 ± 4.67	37.69 ± 4.69	42.88 ± 8.54^**^^##^	<0.01
RVd mid, mm	33.32 ± 10.13	30.89 ± 4.11	31.90 ± 4.68	34.74 ± 5.82^*^^#^	<0.05
TAPSE, mm	18.88 ± 4.58	22.42 ± 7.27	17.83 ± 3.12^**^	18.44 ± 3.72^**^	<0.01
PASP, mmHg	50 (44–63)	47 (41–55)	54 (46–65)^**^	56 (46.5–65)^**^	<0.01
**Left heart disease**					>0.05
Rheumatic	206 (65.0%)	93 (66.9%)	78 (61.9%)	35 (67.3%)	
Degenerative	103 (32.5%)	42 (30.2%)	45 (35.7%)	16 (30.8%)	
Endocarditis	8 (2.5%)	4 (2.9%)	3 (2.4%)	1 (1.9%)	
**Tricuspid repair**					<0.01
Kay procedure	13 (4.1%)	6 (4.3%)	7 (5.6%)	0	
Sorin Sovering	258 (81.4%)	120 (86.3%)	101 (80.2%)	37 (71.2%)	
Edwards MC3	46 (14.5%)	13 (9.4%)	18 (14.3%)	15 (28.8%)	
**Concomitant surgery**					
MVR	167	63	61	33	
AVR	9	4	3	2	
DVR	107	47	45	15	
MVP	45	22	16	7	
CABG	4	0	1	1	
COX-MAZE	5	3	1	1	

*BMI, body mass index; BSA, body surface area; NYHA, New York Heart Association; NT-BNP, N-terminal pro-brain natriuretic peptide; AF, atrial fibrillation; LVDd, left ventricular dimension in diastole; LVDs, left ventricular dimension in systole; LA, left atrial dimension; EF, ejection fraction; FS, shortening fraction; RA S-L, septal-lateral diameter of right atrium in apical four-chamber view; RA S-I, supero-inferior diameter of right atrium in apical four-chamber view; TV S-L, septo-lateral diameter of tricuspid annulus; TV A-P, antero-posterior diameter of tricuspid annulus; RVd mid, middle right ventricular diameter in diastole; TAPSE, tricuspid annular plane systolic excursion; PASP, pulmonary artery systolic pressure; AF, atrial fibrillation; MVR, mitral valve replacement; MVP, mitral valve repair; AVR, aortic valve replacement; DVR, double valve replacement; CABG, coronary artery bypass grafting; ASD, atrial septal defect*.

*Comparison between group 1 and group 2 or group 3:*

* *p < 0.05,*

** *p < 0.01*.

*Comparison between group 2 and group 3:*

# *p < 0.05,*

## *p < 0.01*.

### Assessment of Tricuspid Annulus Dilation

Intraoperative TA data are summarized in Table 2. We observed that each TA segment dilated with an increase in FTR severity (Figure 1). In the course of TA dilation from mild to severe FTR, the anterior annulus dilated ≈13.4%, the posterior annulus dilated ≈17.0%, and the septal annulus dilated ≈11.4%. Considering the difficulty in identifying the anteroposterior commissure in some cases, the ratio between the anteroposterior and septal annuli was calculated, which remained stable during the course of TA dilation. All these findings strongly suggested that each TA segment including septal annulus dilated in the course of TA dilation.

**Table 2:** Tricuspid annulus obtained by intraoperative measurement.

	**Overall (*n* = 317)**	**Mild FTR (*n* = 139)**	**Moderate FTR (*n* = 126)**	**Severe FTR (*n* = 52)**	***P*** **-value**
Anterior annulus, mm	38.44 ± 8.02	36.71 ± 6.30	38.21 ± 8.35^*^	41.63 ± 9.20^**^^##^	<0.01
Posterior annulus, mm	35.13 ± 7.76	33.31 ± 6.94	35.56 ± 7.63^**^	38.98 ± 8.70^**^^#^	<0.01
Septal annulus, mm	40.04 ± 6.62	38.11 ± 5.28	39.76 ± 6.90^**^	42.46 ± 7.50^**^^#^	<0.01
A+P, mm	73.58 ± 13.09	70.02 ± 10.64	73.77 ± 13.14^*^	80.61 ± 14.63^**^^##^	<0.01
Anterior index, mm/m^2^	23.05 ± 5.29	21.83 ± 3.96	22.82 ± 4.85	25.67 ± 7.35^**^^##^	<0.01
Posterior index, mm/m^2^	20.96 ± 4.76	19.81 ± 4.36	21.30 ± 4.54^**^	23.28 ± 5.43^**^^#^	<0.01
Septal index, mm/m^2^	23.92 ± 4.36	22.72 ± 3.92	23.78 ± 4.07^**^	25.49 ± 5.19^**^^#^	<0.01
Anterior annulus%	33.77 ± 4.12	33.92 ± 4.02	33.66 ± 4.26	33.83 ± 4.00	>0.05
Posterior annulus%	30.83 ± 4.47	30.68 ± 4.44	31.32 ± 4.33	31.67 ± 4.95	>0.05
Septal annulus%	35.40 ± 4.22	35.40 ± 4.15	35.02 ± 4.13	34.50 ± 4.62	>0.05
A+P%	64.60 ± 4.22	64.60 ± 4.15	64.98 ± 4.13	66.04 ± 4.62	>0.05
TAC, mm	113.62 ± 16.91	108.13 ± 13.02	113.53 ± 17.40^**^	123.07 ± 18.11^**^^##^	<0.01
TACI, mm/m^2^	67.80 ± 11.07	64.35 ± 9.33	67.90 ± 10.20^**^	74.45 ± 13.26^**^^##^	<0.01

*A+P, the length of anterior plus posterior annulus; Anterior/ Posterior/ Septal index, anterior/ posterior/ septal annulus divided by body surface area; Anterior/ Posterior/ Septal annulus%, the ratio of anterior/ posterior/ septal annulus to the tricuspid annulus circumference; A+P%, the ratio of anterior plus posterior annulus to the tricuspid annulus circumference; TAC, tricuspid annulus circumference; TACI, tricuspid annulus circumference index (circumference/ body surface area). Comparison between group 1 and group 2 or group 3:*

* *p < 0.05,*

** *p < 0.01 Comparison between group 2 and group 3:*

# *p < 0.05,*

## *p < 0.01*.

**Figure 1:**
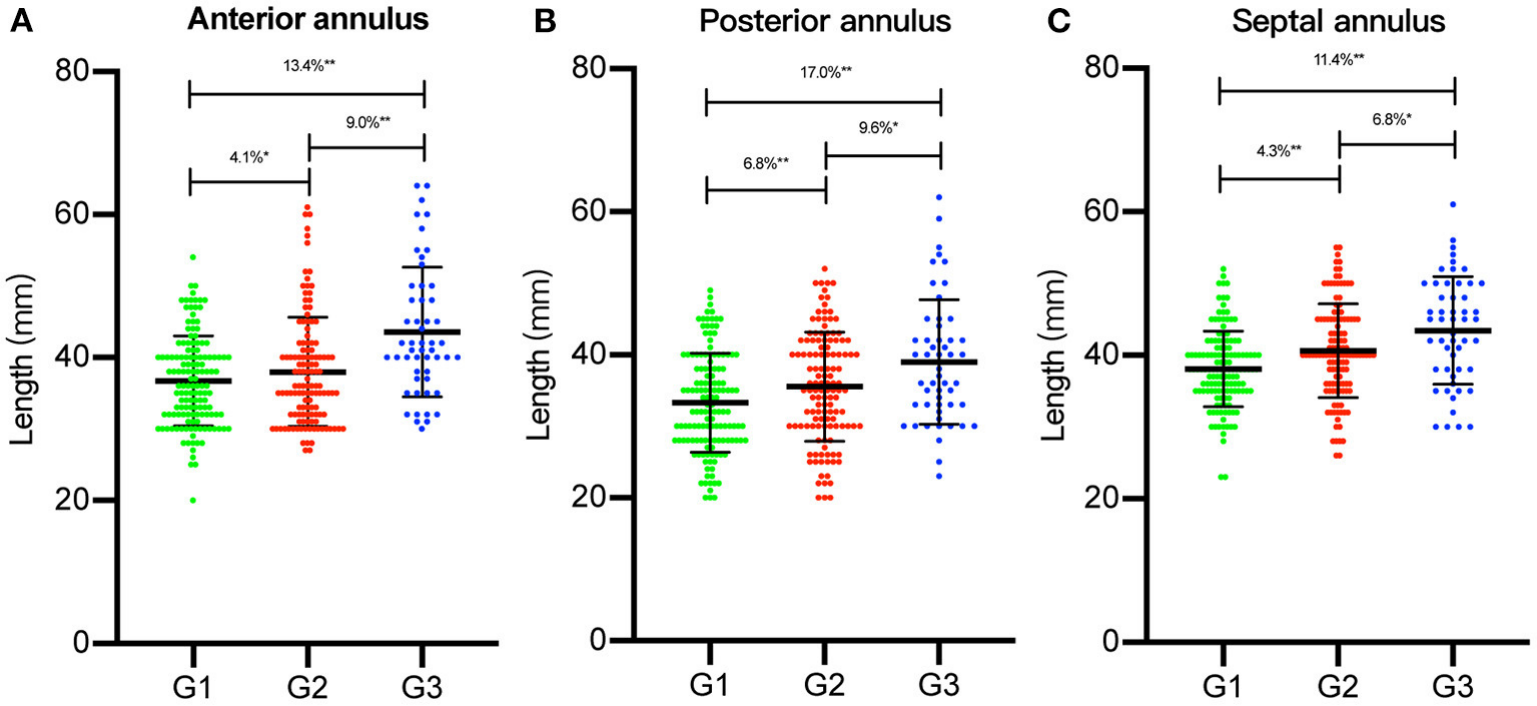
The length of each segment of tricuspid annulus (TA) was measured intraoperatively. Each segment of TA [**(A)** anterior annulus; **(B)** posterior annulus; **(C)** septal annulus] was compared between three groups [group 1 (G1), group 2 (G2), and group 3 (G3)]. The percentage of dilation of each segment of TA was illustrated. *p < 0.05, **p < 0.01.

### Analysis of Factors Correlated With Preoperative Severe FTR

**Figure 2:**
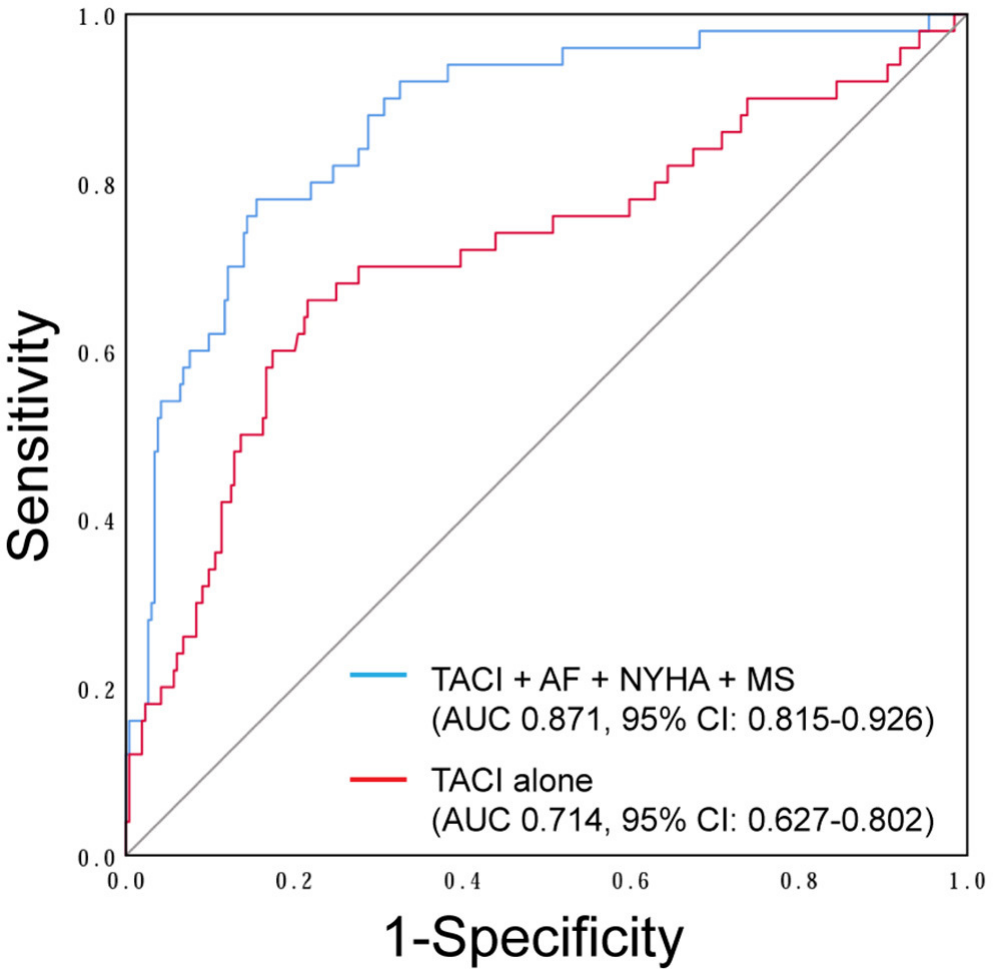
Receiver-operator characteristic curve showing the tricuspid annulus circumference index (TACI) (red line) and combined multivariate analysis model (blue line) including the TACI, NYHA classification, preoperative atrial fibrillation (AF), and preoperative mitral stenosis (MS) as the independent predictors of preoperative severe functional tricuspid regurgitation (FTR).

Age, preoperative AF, NYHA classification, NT-BNP, total bilirubin, creatinine, indices of the right ventricle (RV) and TA geometry, PASP, left atrium (LA) dimension, and etiology of mitral valve disease were found to be highly associated with severe FTR on univariate analysis (Table 3). Multivariate analysis revealed that the tricuspid annulus circumference index (TACI) [odds ratio (OR) 1.064, *p* < 0.01)], NYHA classification (NYHA III: OR 3.027, *p* < 0.01; NYHA IV: OR 9.410, *p* < 0.01), preoperative AF (OR 6.018, *p* < 0.01), and preoperative mitral stenosis (OR 3.165, *p* < 0.01) were independent predictors of severe preoperative FTR (Table 3). Using ROC curves, we observed that the sensitivity and specificity for predicting preoperative severe FTR were 66.0 and 78.0%, respectively, for a TACI of >72.95 mm/m^2^ (area under the curve [AUC]: 0.714, 95% confidence interval [CI]: 0.627–0.802, *p* < 0.01). Moreover, when the TACI was combined with NYHA classification, preoperative mitral stenosis, and preoperative AF, the sensitivity and specificity for predicting preoperative FTR increased to 78.0 and 84.5%, respectively (AUC: 0.871, 95% CI: 0.815–0.926, *p* < 0.01) (Figure 2).

**Table 3:** Univariate and multivariate analysis of risk factors of severe FTR.

	**Univariate analysis**			**Multivariate analysis**		
	**β**	**95% CI**	***P*** **-value**	**β**	**95% CI**	***P*** **-value**
**Preoperative severe FTR**						
Age, per year	0.052	(1.023–1.085)	<0.01			
**NYHA**						
III	1.474	(1.908–9.988)	<0.01	1.108	(1.201–7.629)	<0.05
IV	2.994	(7.387–54.012)	<0.01	2.242	(2.820–31.404)	<0.05
NT-BNP, per 100 pg/mL	0.036	(1.000–1.001)	0.068			
TB, per μmol/L	0.120	(1.081–1.175)	<0.01			
Cr, per μmol/L	0.012	(1.004–1.021)	<0.01			
Preoperative AF	1.882	(2.530–17.049)	<0.01	1.795	(1.889–19.170)	<0.01
LA, per mm	0.038	(1.013–1.065)	<0.01			
RA S-L, per mm	0.094	(1.056–1.144)	<0.01			
RA S-I, per mm	0.090	(1.055–1.134)	<0.01			
TV A-P, per mm	0.185	(1.091–1.326)	<0.01			
TV S-L, per mm	0.253	(1.166–1.422)	<0.01			
PASP, per mmHg	0.18	(0.998–1.038)	0.084			
Anterior annulus, per mm	0.086	(1.051–1.129)	<0.01			
Anterior index, per mm/m^2^	0.144	(1.092–1.220)	<0.01			
Posterior annulus, per mm	0.076	(1.037–1.123)	<0.01			
Posterior index, per mm/m^2^	0.120	(1.057–1.204)	<0.01			
Septal annulus, per mm	0.092	(1.047–1.147)	<0.01			
Septal index, per mm/m^2^	0.135	(1.066–1.229)	<0.01			
TAC, per mm	0.052	(1.034–1.073)	<0.01			
TACI, per mm/m^2^	0.075	(1.047–1.109)	<0.01	0.062	(1.027–1.103)	<0.01
Rheumatic valve disease	0.498	(0.986–2.990)	0.089			
Mitral stenosis	0.986	(1.404–5.128)	<0.01	1.153	(1.379–7.299)	<0.01
Mitral regurgitation	0.640	(1.036–3.472)	<0.05			
**Postoperative recurrent FTR**						
Age, per year	0.060	(1.024–1.102)	<0.01			
BSA, per m^2^	−2.709	(0.005–0.959)	<0.05			
Preoperative FTR			<0.01			
Moderate FTR	1.543	(1.304–16.768)	<0.05			
Severe FTR	2.463	(3.336–41.275)	<0.01	2.548	(1.961–45.955)	<0.05
Postoperative AF	1.026	(1.055–7.372)	<0.05			
TV S-L, per mm	0.046	(0.988–1.110)	0.081			
RA S-I, per mm	0.038	(1.009–1.069)	<0.05			
RA S-L, per mm	0.035	(1.005–1.067)	<0.05			
Anterior annulus, per mm	0.039	(1.000–1.080)	0.050			
Anterior index, per mm/m^2^	0.078	(1.028–1.138)	<0.01			
Posterior index, per mm/m^2^	0.078	(1.005–1.163)	<0.05			
Septal annulus, per mm	0.047	(0.997–1.109)	0.092			
Septal index, per mm/m^2^	0.091	(1.013–1.183)	<0.05			
TAC, per mm	0.023	(1.003–1.045)	<0.05			
TACI, per mm/m^2^	0.044	(1.014–1.077)	<0.01	0.044	(1.014–1.077)	<0.01
Rheumatic valve disease	0.332	(0.973–2.038)	0.087			

*NYHA, New York Heart Association; NT-BNP, N-terminal pro-brain natriuretic peptide; TB, total bilirubin; Cr, creatinine; AF, atrial fibrillation; LVDd, left ventricular dimension in diastole; LVDs, left ventricular dimension in systole; LA, left atrial dimension; EF, ejection fraction; RA S-L, septal-lateral diameter of right atrium in apical four-chamber view; RA S-I, supero-inferior diameter of right atrium in apical four-chamber view; TV S-L, septo-lateral diameter of tricuspid annulus; TV A-P, antero-posterior diameter of tricuspid annulus; RVd mid, middle right ventricular diameter in diastole; TAPSE, tricuspid annular plane systolic excursion; PASP, pulmonary artery systolic pressure; Anterior/ Posterior/ Septal index, anterior/ posterior/septal annulus divided by body surface area; TAC, tricuspid annulus circumference; TACI, tricuspid annulus circumference index (circumference/ body surface area)*.

### Analysis of Factors Correlated With Postoperative FTR Recurrence

**Figure 3:**
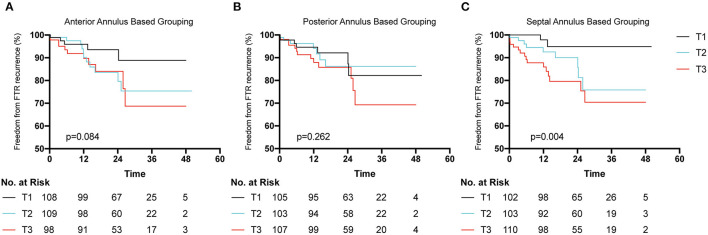
Freedom from postoperative functional tricuspid regurgitation (FTR) recurrence according to the length of each segment of tricuspid annulus. Patients were grouped into tertiles (T1 shortest length and T3 longest length). **(A)** Patients were trisected based on the length of anterior annulus. **(B)** Patients were trisected based on the length of posterior annulus. **(C)** Patients were trisected grouped based on the length of septal annulus.

Univariate analysis identified 15 variables that correlated substantially with FTR recurrence during follow-up (Table 3). Interestingly, neither the methods of tricuspid repair nor size of the tricuspid ring correlated with FTR recurrence. Of these variables, preoperative severe FTR (OR 9.494, *p* < 0.05) and the TACI (OR 1.045, *p* < 0.01) independently correlated with FTR recurrence in Cox regression analysis. Patients were grouped into tertiles (T) based on the length of each annulus segment (T1, shortest; T3, longest). KM survival analysis demonstrated no significant difference between T1 and T3 when the patients were grouped based on the length of the anterior or posterior annulus (Figures 3A,B), while a significant difference was detected based on the length of the septal annulus (Figure 3C).

## Discussion

To the best of our knowledge, this was the first study to evaluate the dilation course of each TA segment in FTR from a surgical perspective. The primary finding of our study was that each TA segment, including the septal segment, could dilate in FTR ranging from 10 to 20% (posterior, 17%; anterior, 13.4%; septal, 11.4%), which was significantly lower than the one reported previously (posterior, 80%; anterior, 40%; septal, 20%) (5). Second, we observed that the length of the septal annulus demonstrated a higher sensitivity to postoperative FTR recurrence than that of the anterior or posterior annulus. Thus, we highlight the potential benefits of septal plication in tricuspid repair to achieve better long-term outcomes.

Cardiovascular surgeons often refer to Carpentier's technique of tricuspid repair, especially tricuspid annuloplasty using prosthetic rings (7). This technique was derived from the work of Deloche et al. who proposed the tricuspid dilation theory which stated that tricuspid dilation occurred primarily at the posterior annulus, followed by the anterior annulus, and finally at the septal annulus (5, 6). Few cardiovascular surgeons recognized that the data supporting this theory were derived from post-mortem studies of 10 healthy and 15 rheumatic hearts without any other baseline characteristics (6). Currently available prosthetic tricuspid rings, including Edwards MC3 (15), Carpentier-Edwards Physio Tricuspid, Medtronic TriAd, and Medtronic Contour 3D (16), are designed based on this theory and are implanted according to Carpentier's technique (7). However, markers vary among the prosthetic rings (8). Furthermore, it is difficult to select the ring size in cases with a dilated septal annulus, which is larger than the largest obturator size. The gold standard technique for tricuspid annuloplasty and ideal size for the tricuspid ring remain unclear.

Recently, echocardiographic studies have reported that different mechanisms of FTR would lead to different geometric changes in TA (17). Compared with ventricular FTR, atrial FTR is more dilated and has a more posteriorly displaced annulus (18), while pulmonary hypertension-related FTR correlates more significantly with leaflet tethering during RV elongation compared with atrial FTR (19). All these studies focused on the echocardiographic geometry and orientation of tricuspid dilation in FTR. However, few studied focused on the dilation of each TA segment from a surgical perspective. In this study, we investigated the course of TA dilation in FTR by measuring the length of each TA segment during open-heart surgery, and provided evidence to support the notion that the septal annulus could dilate in FTR.

### Course of TA Dilation in FTR

Based on the echocardiographic study, TA demonstrated increased annular area, planarity, and circularity with the development of FTR. According to our measurements, each TA segment dilated with an increase in the FTR severity. Compared with group 1, group 2 demonstrated increased dilation of the anterior, posterior, and septal annuli of ≈4.1, 6.8, and 4.1%, respectively. Similarly, compared with group 2, the anterior, posterior, and septal annuli in group 3 were significantly more dilated by ≈9.0, 9.6, and 6.8%, respectively. We observed that from mild to severe FTR, each TA segment dilated significantly ranging from 10 to 20%. Specifically, the posterior annulus was dilated with the largest ratio of ≈17.0%, followed by the anterior (13.4%) and septal annuli (11.4%). Such tricuspid annulus dilation is considered almost proportionate. Based on our data, we confirmed Deloche's findings that the posterior annulus dilated with the largest ratio in FTR. However, there was no significant difference in the dilation ratio between each TA segment, as reported previously (80, 40, and 20% for the posterior, anterior, and septal annuli, respectively) (5).

The reason for the above findings, especially septal annulus dilation, which are contrary to Deloche's theory, remains unknown. The possible reasons are as follows: first, Deloche's study had a small sample size with unknown inter-individual variations. Moreover, this study was executed with heart immersion in formalin fixation, which was known to cause morphometric changes in the preserved tissue, including the TA (20, 21).

Second, Kawada et al. stated that the posteroseptal commissural point was located on the free wall of the RV rather than on the interventricular septum (11). In addition, according to Seccombe et al. it was not unusual for the septal leaflet to “wrap around” onto the posterior portion of the free wall by 1 cm (22). These studies suggested that only the partial septal annulus was fixed on the fibrous trigones, while the remaining part would easily and synchronously dilate with the free wall of the RV in FTR, which explains the lower degree of dilation than the posterior annulus.

Third, in addition to the muscular part of the septal annulus (the part attached to the free wall of the RV), the fibrous part of the septal annulus (the part attached to the interventricular septum) might have also been affected by the dilation of the RV with pulmonary hypertension caused by any reason (23). Similar to the septal annulus, the fibrous portion of the mitral annulus has traditionally been considered a static part that does not dilate in functional mitral regurgitation. However, it was revealed that the fibrous part of the mitral annulus was proportionately dilated in patients with cardiomyopathy (24) and in animal models of biventricular failure (25). Therefore, it is not unreasonable to expect that the septal annulus of the tricuspid valve would dilate in FTR and a dilated RV.

Fourth, differences among the races and disease spectra should be considered. To the best of our knowledge, the studies which proposed notions contrary to Deloche's theory and Carpentier's technique were predominantly from Japan (10). Kondoh and colleagues introduced a new ring annuloplasty in the case of an extremely dilated septal annulus of 60 mm (10). Isomura et al. proposed a technique of septal plication in patients with FTR and significantly improved the intraoperative repair success rate from 62 to 93% (9). Two studies specified that the markers on tricuspid rings vary among manufacturers and suggested new techniques for tricuspid ring annuloplasty (11, 12). The underlying explanation remains unknown and requires further investigation.

### The Association Between the Size of TA and the Severity of FTR and Postoperative FTR Recurrence

The severity of FTR fluctuates greatly and is affected by various factors, such as the volume of systemic circulation, heart rate and rhythm, conscious state, and pulmonary artery pressure. Based on the European Society of Cardiology/European Association for Cardiothoracic Surgery and ACC/AHA guidelines, only a diameter of TA ≥40 mm or TA/BSA >21 mm/m^2^ is considered an indication for tricuspid repair in patients undergoing left heart surgery (14, 26). Thus, it is evident that echocardiographic evaluation of FTR is not comprehensive and accurate, and reliable assessment of FTR continues to be a challenge. Our study reported that the TACI was an independent risk factor for preoperative FTR and postoperative FTR recurrence. In a study by Xu Meng et al., a tricuspid annulus circumference (TAC) of >11.45 cm and a TACI of >7.07 cm/m^2^ were considered important factors of TA dilation in FTR (27). Our study concluded that a TAC of >11.35 cm and a TACI of >72.95 mm/m^2^ were the risk factors for preoperative severe FTR. Thus, we highlight the importance of the measurement of TA intraoperatively, which could provide valuable and accurate evaluation of FTR severity.

Several factors have been considered to correlate significantly with postoperative FTR recurrence, such as preoperative severity of FTR, postoperative AF, size of the right atrium (RA) or RV, heart function, permanent pacemaker implantation, pulmonary hypertension, leaflet tethering, and rheumatic etiology of left heart disease (28, 29). This study reported that the preoperative severity of FTR and TACI independently correlated with postoperative FTR recurrence. Moreover, we observed that the septal annulus could dilate in FTR, and its length displayed higher sensitivity for predicting postoperative FTR recurrence than that of the anterior or posterior annulus. The possible reasons for such a result might be: (1) TA dilation usually accompanies enlargement of the right heart and decreased RV function. The longer the TAC is, the larger the right heart becomes, and the RV function worsens, which is attributed to recurrent regurgitation; (2) the entire posterior and major anterior annuli are attached to the free wall of the RV, which is prone to dilation when RV overload occurs. However, when the septal annulus is partially attached to the interventricular septum, it is less prone to dilation. Once the septal annulus is dilated, it is more likely that RV remodeling occurs and deterioration of RV starts, which explains why the length of the septal annulus displayed higher sensitivity for predicting postoperative FTR recurrence.

### Clinical Implication for Tricuspid Annuloplasty

The concept of tricuspid dilation is as follows: dilation of the tricuspid annulus chiefly occurs in the posterior and anterior annuli. Currently available tricuspid rings have markers indicating the suturing position of the anteroposterior and septoposterior commissures. Such rings are designed especially for posterior annulus plication; however, based on our data, which is in accordance with other studies, we observed that each TA segment could dilate in FTR. These findings have significant clinical implications, as the current tricuspid ring might not entirely support the septal annulus, resulting in FTR recurrence. Thus, we highlight the potential benefits of septal annulus plication. We previously reported our 3-J technique for tricuspid annuloplasty to improve the FTR outcome (30). The early results of our 3-J technique were favorable, and further research is in progress to confirm our concept.

### Limitations

First, this was not a randomized controlled study; whether a concomitant tricuspid repair should be performed in patients with trivial or mild FTR while undergoing left heart surgery was decided by surgeons, which would cause selection bias. Second, not all patients were entirely followed up due to poor medical conditions of some patients who cannot return for a follow-up and no available international medical data-sharing platform. Finally, echocardiographic evaluation of the right heart and TV function was not comprehensive, as some parameters such as RA area, RA volume, and TV tenting height and area, were not routinely measured in our center.

### Comment

Each TA segment could dilate with FTR severity (from trivial or mild to severe FTR) ranging from 10 to 20%, which varies from the previously reported results. The TACI is independently associated with severe preoperative FTR and postoperative FTR recurrence. The length of the septal annulus displays higher sensitivity to postoperative FTR recurrence than the anterior or posterior annulus. Based on our findings, septal plication should be performed during tricuspid annuloplasty to obtain favorable long-term results.

## Data Availability Statement

The original contributions presented in the study are included in the article/Supplementary Material, further inquiries can be directed to the corresponding author/s.

## Ethics Statement

The retrospective use of the database and this study were approved by the Institutional Review Board and Ethics Committee of the First Affiliated Hospital, College of Medicine, Zhejiang University.

## Author Contributions

Study conception and design was performed by PT, LM, and YN. The echocardiographic data was collected by SY and YC. The clinical data was collected by XD. PT drafted the manuscript and was revised by YZ. All authors confirmed the final version of the paper, read, and approval the final manuscript.

## Funding

This study was funded by Key Research and Development Program, Zhejiang Province, Project Number: 2019C03008 and funded by Public Welfare Technology Application Research Project, Zhejiang Province, Project Number: LGF19H020012.

## Conflict of Interest

The authors declare that the research was conducted in the absence of any commercial or financial relationships that could be construed as a potential conflict of interest.

## Publisher's Note

All claims expressed in this article are solely those of the authors and do not necessarily represent those of their affiliated organizations, or those of the publisher, the editors and the reviewers. Any product that may be evaluated in this article, or claim that may be made by its manufacturer, is not guaranteed or endorsed by the publisher.

## Supplementary Material

Supplementary Figure 1 **(A–D)** Intraoperative photographs show that the length of the septal annulus is significantly larger than the length between the notches on the obturator. The notch (red arrows) on the obturator, which should be aligned to the commissure (yellow arrows) of the septal annulus according to the manufacturer's protocol.

## Abbreviations

FTR: functional tricuspid regurgitation
AF: atrial fibrillation
TA: tricuspid annulus
TV: tricuspid valve
ASD: atrial septal defect
CABG: coronary artery bypass grafting
ROC: receiver-operator characteristic
AUC: area under the curve
NYHA: New York Heart Association
NT-BNP: N-terminal pro-brain natriuretic peptide
PASP: pulmonary arterial systolic pressure
TAPSE: tricuspid annular plane systolic excursion
RV: right ventricle
RA: right atrium
KM: Kaplan–Meier
TAC: tricuspid annulus circumference
TACI: tricuspid annulus circumference index
VC: vena contracta.

## References

[1] Kim WK Kim SE Yoo JS Jung JH Kim DH Kim JB. Impact of valve repair on mild tricuspid insufficiency in rheumatic mitral surgery. J Thorac Cardiovasc Surg. (2021) 2:S0022-5223(21)00878-3. 10.1016/j.jtcvs.2021.05.033 34154801

[2] Nath J Foster E Heidenreich PA. Impact of tricuspid regurgitation on long-term survival. J Am Coll Cardiol. (2004) 43:405–9. 10.1016/j.jacc.2003.09.036 15013122

[3] Chen Y Chan YH Wu MZ Yu YJ Lam YM Sit KY. Prevalence and prognostic importance of massive tricuspid regurgitation in patients undergoing tricuspid annuloplasty with concomitant left-sided valve surgery: a study on rheumatic valvular heart disease. Front Cardiovasc Med. (2022) 9:686208. 10.3389/fcvm.2022.686208 35155624 PMC8829045

[4] Belluschi I Del Forno B Lapenna E Nisi T Iaci G Ferrara D. Surgical techniques for tricuspid valve disease. Front Cardiovasc Med. (2018) 5:118. 10.3389/fcvm.2018.00118 30234129 PMC6127626

[5] Deloche A Guerinon J Fabiani JN Morillo F Caramanian M Carpentier A. [Anatomical study of rheumatic tricuspid valve diseases: application to the study of various valvuloplasties]. Ann Chir Thorac Cardiovasc. (1973) 12:343–9. 4771714

[6] Acar C Perier P Fontaliran F Deloche A Carpentier A. Anatomical study of the tricuspid valve and its variations. Surg Radiol Anat. (1990) 12:229–30. 10.1007/BF01624529 2287991

[7] Carpentier A. Cardiac valve surgery–the “French correction”. J Thorac Cardiovasc Surg. (1983) 86:323–37. 10.1016/S0022-5223(19)39144-5 6887954

[8] Mathur M Malinowski M Timek TA Rausch MK. Tricuspid annuloplasty rings: a quantitative comparison of size, nonplanar shape, and stiffness. Ann Thorac Surg. (2020) 110:1605–14. 10.1016/j.athoracsur.2020.02.064 32251659 PMC11040511

[9] Isomura T Hirota M Hoshino J Fukada Y Kondo T Takahashi. Tricuspid annuloplasty with the MC3 ring and septal plication technique. Asian Cardiovasc Thorac Ann. (2015) 23:5–10. 10.1177/0218492314529953 24682337

[10] Kondoh H Hatsuoka S Shintani H. New ring annuloplasty for extremely dilated tricuspid valve annulus: plication to physiologic septal segment size and over-reduction of posterior segment. Tex Heart Inst J. (2009) 36:327–30. 19693308 PMC2720292

[11] Kawada N Naganuma H Muramatsu K Ishibashi-Ueda H Bando K Hashimoto. Redefinition of tricuspid valve structures for successful ring annuloplasty. J Thorac Cardiovasc Surg. (2018) 155:1511–19 e1. 10.1016/j.jtcvs.2017.12.045 29366576

[12] Sakon Y Murakami T Fujii H Takahashi Y Morisaki A Yamane K. New insight into tricuspid valve anatomy from 100 hearts to reappraise annuloplasty methodology. Gen Thorac Cardiovasc Surg. (2019) 67:758–64. 10.1007/s11748-019-01092-9 30805826

[13] Pfannmuller B Doenst T Eberhardt K Seeburger J Borger MA Mohr W.. Increased risk of dehiscence after tricuspid valve repair with rigid annuloplasty rings. J Thorac Cardiovasc Surg. (2012) 143:1050–5. 10.1016/j.jtcvs.2011.06.019 21798563

[14] Otto CM Nishimura RA Bonow RO Carabello BA Erwin JP III Gentile F. 2020 ACC/AHA guideline for the management of patients with valvular heart disease: executive summary: a report of the American College of Cardiology/American Heart Association Joint Committee on Clinical Practice Guidelines. Circulation. (2021) 143:e35–71. 10.1161/CIR.0000000000000932 33332149

[15] Filsoufi F Salzberg SP Coutu M Adams DH. A three-dimensional ring annuloplasty for the treatment of tricuspid regurgitation. Ann Thorac Surg. (2006) 81:2273–7. 10.1016/j.athoracsur.2005.12.044 16731167

[16] Ratschiller T Guenther T Guenzinger R Noebauer C Kehl V Gertler R. Early experiences with a new three-dimensional annuloplasty ring for the treatment of functional tricuspid regurgitation. Ann Thorac Surg. (2014) 98:2039–44. 10.1016/j.athoracsur.2014.07.023 25443010

[17] Gercek M Rudolph V. Secondary tricuspid regurgitation: pathophysiology, incidence and prognosis. Front Cardiovasc Med. (2021) 8:701243. 10.3389/fcvm.2021.701243 34368256 PMC8339586

[18] Utsunomiya H Harada Y Susawa H Ueda Y Izumi K Itakura K. Tricuspid valve geometry and right heart remodelling: insights into the mechanism of atrial functional tricuspid regurgitation. Eur Heart J Cardiovasc Imaging. (2020) 21:1068–78. 10.1093/ehjci/jeaa194 32756989

[19] Topilsky Y Khanna A Le Tourneau T Park S Michelena H Suri R. Clinical context and mechanism of functional tricuspid regurgitation in patients with and without pulmonary hypertension. Circ Cardiovasc Imaging. (2012) 5:314–23. 10.1161/CIRCIMAGING.111.967919 22447806

[20] Eckner FA Brown BW Overll E Glagov S. Alteration of the gross dimensions of the heart and its structures by formalin fixation. A quantitative study. Virchows Arch A Pathol Pathol Anat. (1969) 346:318–29. 10.1007/BF00542709 5306088

[21] Holda MK Klimek-Piotrowska W Koziej M Piatek K Holda J. Influence of different fixation protocols on the preservation and dimensions of cardiac tissue. J Anat. (2016) 229:334–40. 10.1111/joa.12469 27031944 PMC4948045

[22] Seccombe JF Cahill DR Edwards WD. Quantitative morphology of the normal human tricuspid valve: autopsy study of 24 cases. Clin Anat. (1993) 6:203–12. 10.1002/ca.980060402

[23] Malinowski M Wilton P Khaghani A Langholz D Hooker V Eberhart L. The effect of pulmonary hypertension on ovine tricuspid annular dynamics. Eur J Cardiothorac Surg. (2016) 49:40–5. 10.1093/ejcts/ezv052 25755186

[24] Hueb AC Jatene FB Moreira LF Pomerantzeff PM Kallas E de Oliveira A.. Ventricular remodeling and mitral valve modifications in dilated cardiomyopathy: new insights from anatomic study. J Thorac Cardiovasc Surg. (2002) 124:1216–24. 10.1067/mtc.2002.125342 12447190

[25] Timek TA Dagum P Lai DT Liang D Daughters GT Tibayan F. Tachycardia-induced cardiomyopathy in the ovine heart: mitral annular dynamic three-dimensional geometry. J Thorac Cardiovasc Surg. (2003) 125:315–24. 10.1067/mtc.2003.80 12579100

[26] Vahanian A Beyersdorf F Praz F Milojevic M Baldus S Bauersachs J. 2021 ESC/EACTS Guidelines for the management of valvular heart disease. Eur Heart J. (2021) 43:561–32. 10.1093/ejcts/ezac209 34931612 PMC9725093

[27] Xu J Han J Zhang H Meng F Luo T Tian B. Risk factors for postoperative recurrent tricuspid regurgitation after concomitant tricuspid annuloplasty during left heart surgery and the association between tricuspid annular circumference and secondary tricuspid regurgitation. BMC Cardiovasc Disord. (2021) 21:50. 10.1186/s12872-021-01870-5 33499803 PMC7836580

[28] Huang Y Dearani JA Lahr BD Stephens EH Madhavan M Cannon BC. Surgical management of transvenous lead-induced tricuspid regurgitation in adult and pediatric patients with congenital heart disease. J Thorac Cardiovasc Surg. (2021) 9:S0022-5223(21)01419-7. 10.1016/j.jtcvs.2021.10.006 34753592

[29] McCarthy PM Szlapka M Kruse J Kislitsina ON Thomas JD Liu M. The relationship of atrial fibrillation and tricuspid annular dilation to late tricuspid regurgitation in patients with degenerative mitral repair. J Thorac Cardiovasc Surg. (2021) 161:2030–40 e3. 10.1016/j.jtcvs.2019.11.098 31952828

[30] Xu H Davies H Zheng J Peng T Ni Y. Modified band annuloplasty technique for functional tricuspid regurgitation repair in patients with grossly dilated annuli: the three-suture junctional continuous suture band annuloplasty technique. J Card Surg. (2019) 34:167–9. 10.1111/jocs.14006 30834563

