# Supplementary figures and images for: Tricuspid Annulus Dilation in Patients With Combined Functional Tricuspid Regurgitation and Left-Heart Valvular Disease: Does Septal Annulus Not Dilate?
Source: Front Cardiovasc Med. 2022 Apr 26;9:889163. doi: 10.3389/fcvm.2022.889163 (PMC9086675; doi:10.3389/fcvm.2022.889163)

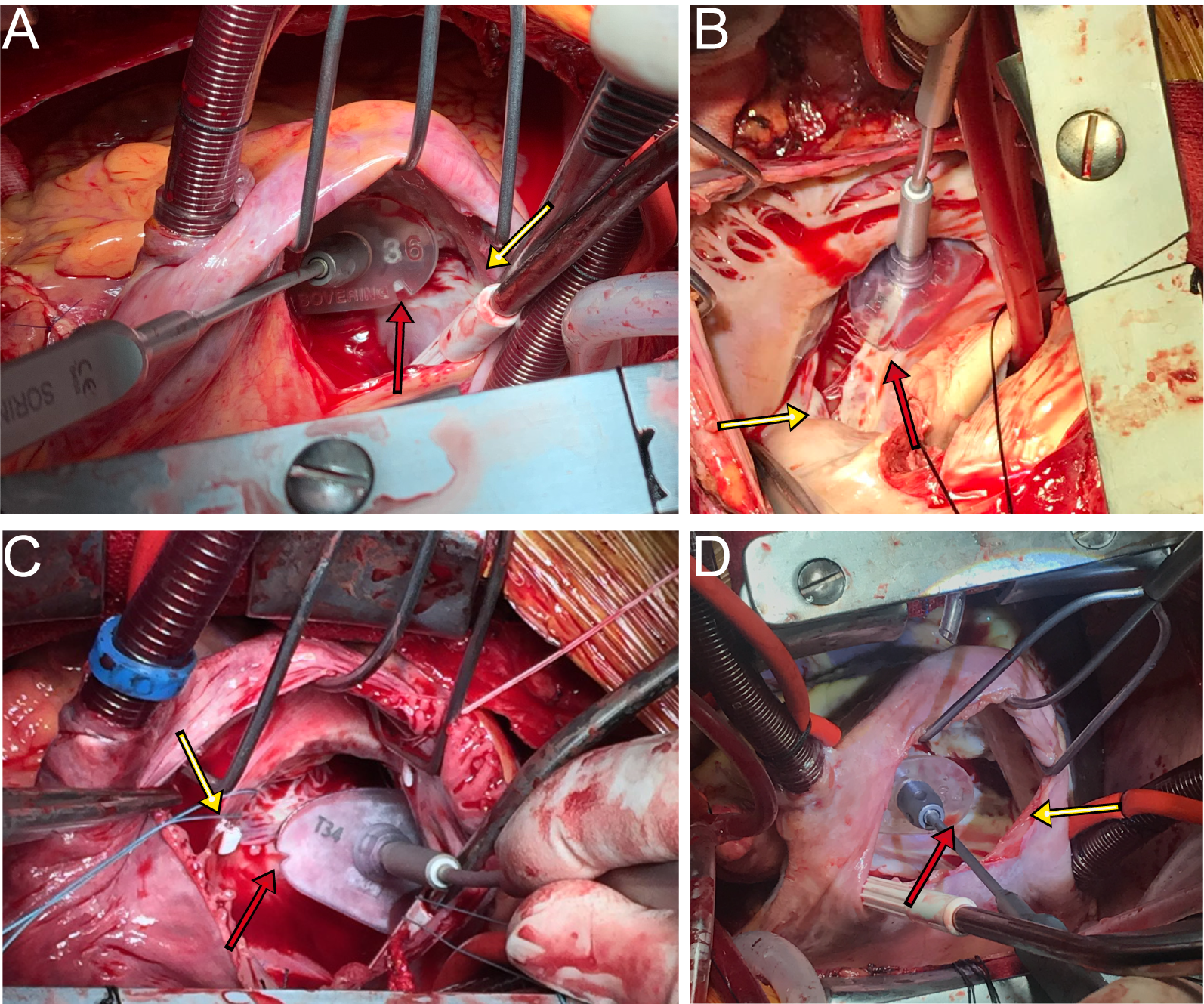

Supplement: Supplementary Figure 1 — (A–D) Intraoperative photographs show that the length of the septal annulus is significantly larger than the length between the notches on the obturator. The notch (red arrows) on the obturator, which should be aligned to the commissure (yellow arrows) of the septal annulus according to the manufacturer's protocol. [file Image_1.TIF]
